# Prognostic Signature of Osteosarcoma Based on 14 Autophagy-Related Genes

**DOI:** 10.3389/pore.2021.1609782

**Published:** 2021-07-16

**Authors:** Wei Qi, Qian Yan, Ming Lv, Delei Song, Xianbin Wang, Kangsong Tian

**Affiliations:** ^1^ Department of West Hospital Orthopaedic Trauma, Zibo Central Hospital, Zibo, China; ^2^ Department of Information Section, Zibo Central Hospital, Zibo, China; ^3^ Department of Eastern Hospital Orthopaedic Trauma, Zibo Central Hospital, Zibo, China

**Keywords:** prognosis, risk score, osteosarcoma, autophagy-related genes, nomogram

## Abstract

**Background:** Osteosarcoma is a common malignancy of bone with inferior survival outcome. Autophagy can exert multifactorial influence on tumorigenesis and tumor progression. However, the specific function of genes related to autophagy in the prognosis of osteosarcoma patients remains unclear. Herein, we aimed to explore the association of genes related to autophagy with the survival outcome of osteosarcoma patients.

**Methods:** The autophagy-associated genes that were related to the prognosis of osteosarcoma were optimized by LASSO Cox regression analysis. The survival of osteosarcoma patients was forecasted by multivariate Cox regression analysis. The immune infiltration status of 22 immune cell types in osteosarcoma patients with high and low risk scores was compared by using the CIBERSORT tool.

**Results:** The risk score model constructed according to 14 autophagy-related genes (ATG4A, BAK1, BNIP3, CALCOCO2, CCL2, DAPK1, EGFR, FAS, GRID2, ITGA3, MYC, RAB33B, USP10, and WIPI1) could effectively predict the prognosis of patients with osteosarcoma. A nomogram model was established based on risk score and metastasis.

**Conclusion:** Autophagy-related genes were identified as pivotal prognostic signatures, which could guide the clinical decision making in the treatment of osteosarcoma.

## Background

Osteosarcoma, also known as osteogenic sarcoma, is considered to be the most common malignancy in bone among children and adolescents [1, 2]. Although the metaphyseal region is the most common location of osteosarcoma, this disease usually progresses rapidly and is prone to metastasis [3, 4]. The prognosis of osteosarcoma is poor and it seriously threatens the life and health of adolescents [5]. Therefore, it is urgent to explore novel targets or signatures for improving the clinical practice of osteosarcoma patients in the future.

Autophagy is a biological process mediated by certain genes, in which the aberrant organelles as well as macromolecules are digested by lysosomes, and it is involved in multiple processes such as cell metabolism, renewal of organelles and intracellular homeostasis maintenance [4, 6]. In recent years, several studies have indicated the relationship of autophagy with the initiation and progression of various diseases, such as cancer and diseases associated with neurodegeneration and immunization [7]. In bladder cancer, autophagy could inhibit the tumorigenesis *via* limiting tissue damage and oncogenic signaling [8]. Furthermore, autophagy could eliminate the accumulation of damaged proteins and organelles, indicating its role in the prevention of tumorigenesis. However, other research has reported that autophagy was essential for enhancing the survival ability of tumor cells and suppressing the necrosis in some cancers, including melanoma and breast cancer [9, 10]. Recycling by autophagy is necessary for the maintenance of energy balance and mitochondrial metabolism for tumor growth and proliferation. In addition, suppression of autophagy is considered as a potential modality for tumor treatment [11]. Autophagy also plays a significant role in osteosarcoma. It has been proved that autophagy is induced in osteosarcoma, and several intermediates are implicated in this process [12]. Autophagy could be promoted by the increased expression of high mobility group box 1, contributing to the drug resistance during the treatment of osteosarcoma [13]. Sun et al. reported that the silence of autophagy-related gene 5 reduced the malignancy of osteosarcoma with anti-oncogenic effects [14]. Moreover, Liu et al. found that the expression of autophagy-related 4B was obviously elevated, which accelerated osteosarcoma development and suppressed the apoptosis of osteosarcoma cells [15]. Consequently, analysis and identification of autophagy-related genes are helpful to improve our knowledge on the association of autophagy with osteosarcoma.

Herein, we analyzed 210 genes associated with autophagy and identified 14 optimized autophagy-associated genes related to the survival outcome of osteosarcoma patients. A death risk model based on those 14 autophagy-related genes could effectively predict the prognosis of osteosarcoma patients. Finally, we established the nomogram model by including independent factors of prognosis (risk score and metastasis) and demonstrated its better performance in predicting the long-term prognosis of osteosarcoma patients.

## Methods

### Data Sources

We downloaded the mRNA expression profiles of 88 osteosarcoma patients with their corresponding clinical information from the Therapeutically Applicable Research to Generate Effective Treatments (TARGET, https://ocg.cancer.gov/programs/target) database. Among them, 85 patients had complete survival information, whose clinicopathological features are depicted in Table 1. Moreover, by using osteosarcoma and survival as keywords in the Gene Expression Omnibus (GEO, https://www.ncbi.nlm.nih.gov/geo/) database, we obtained the mRNA expression information and clinical information of GSE21257 [16] and GSE16091 [17]. Through the SVA package of R software, the batch effect was removed between different datasets. The two datasets were combined to verify the prognostic model. GSE21257 included 53 osteosarcoma samples, and the mRNA expression profile data was detected using Illumina human-6 v2.0 expression beadchip. GSE16091 consisted of 34 osteosarcoma samples, and mRNA expression profile was quantified by Affymetrix Human Genome U133A Array. The clinicopathological characteristics of osteosarcoma patients in GEO datasets were shown in Supplementary Table S1. The mRNA expression data that was previously normalized was used in our study. In addition, we selected the 210 genes associated with autophagy by referring to the previous study [18] and Human Autophagy Database (HADb, www.autophagy.lu/project.html), whose details are displayed in the attached Supplementary Table S2.

**TABLE 1:** Clinicopathological characteristics of OS patients from TARGET database.

Characteristics		Patients (N = 85)	
		No.	%
Sex	Female	37	43.53
	Male	47	55.29
	Unknown	1	1.18
Age	≤14 (Median)	44	51.76
	>14 (Median)	40	47.06
	Unknown	1	1.18
Race	White	51	60.00
	Asian	6	7.06
	Black or African American	7	8.24
	Unknown	21	24.71
Disease at diagnosis	Metastatic disease	21	24.71
	Non-metastatic disease	63	74.12
	Unknown	1	1.18
Primary tumor site	Arm/hand	6	7.06
	Leg/foot	76	89.41
	Pelvis	2	2.35
	Unknown	1	1.18
Vital status	Dead	27	31.76
	Alive	58	68.24

### Cluster Analysis

According to the mRNA expression levels of the 210 genes related to autophagy, the samples were clustered by using factoextra package in R software (https://CRAN.R-project.org/package=factoextra), followed by principal component analysis (PCA).

### LASSO Cox Regression Analysis

Univariate Cox regression analysis was carried out basing on the mRNA expression levels of 210 genes associated with autophagy, and by using the threshold of *p* < 0.05, the autophagy-associated genes that were related to the survival outcome of osteosarcoma patients were selected. Subsequently, LASSO Cox regression analysis was carried out with glmnet package in R software [19]. In the LASSO regression model, the lambda value which corresponded to the minimum value of partial likelihood deviance was considered as the best one, and the best tuning parameter lambda was used to screen the genes associated with autophagy that showed a significant relationship with the survival outcome of osteosarcoma.

$$Risk\;score=\sum_{i=1}^{n}Coef_{i}*x_{i}$$ (1)

Among them, the risk coefficients of all factors were computed using LASSO Cox model and expressed as *Coef*_*i*_, *X*_*i*_ represented the mRNA expression levels of factors. We confirmed the optimal cutoff value of the risk score *via* survival and survminer packages in R software and bilateral log rank test, and then stratified these osteosarcoma patients into high and low risk groups by the above cutoff value.

### Survival Analysis

The overall survival (OS) rate of different groups was assessed by using survival and survminer packages in R software. R language survival ROC package [20] was applied to draw the time-dependent ROC curve. The multivariate Cox regression model was constructed to verify whether risk score was an independent signature for osteosarcoma prognosis after adjusting for multiple factors. The OS of different groups were evaluated by using the Kaplan-Meier method [21] followed by OS comparison *via* log-rank test. The divergences in infiltrating immune cells between different groups were analyzed by Wilcoxon signed-rank test [22] using *p* value less than 0.05 as the threshold. All analyses were carried out by R software (version 3.5.2).

### Immune Cell Infiltration Proportion Analysis

The relative ratio of 22 immune cell types was computed using CIBERSORT [23], which characterized the composition of immune infiltration cells by deconvolution algorithm using the preset 547 barcode genes on the base of gene expression matrix. We set the sum of immune cell ratios as 1 for all samples.

### Gene Set Enrichment Analysis

GSEA (version 4.0.3) [24] was used for gene set enrichment analysis with c2.cp.kegg.v7.0. symbols derived from Molecular Signatures Database (MSigDB) as the gene set. The significantly enriched KEGG pathway was screened with *p* < 0.05 as the threshold.

### Nomogram Model Construction

Nomogram is an important approach for the prediction of cancer prognosis [25]. Herein, the nomogram model was established using the rms package in R software, based on the independent factors for osteosarcoma prognosis obtained from multivariate Cox regression analysis, in an attempt to forecast the 1-year, 3-years, and 5-years OS of osteosarcoma patients. Furthermore, the calibration curve was plotted to estimate the divergence between the predicted and actual OS probabilities.

## Results

### Autophagy-Related Genes Distinguished Osteosarcoma Patients With Different Prognoses

**FIGURE 1:**
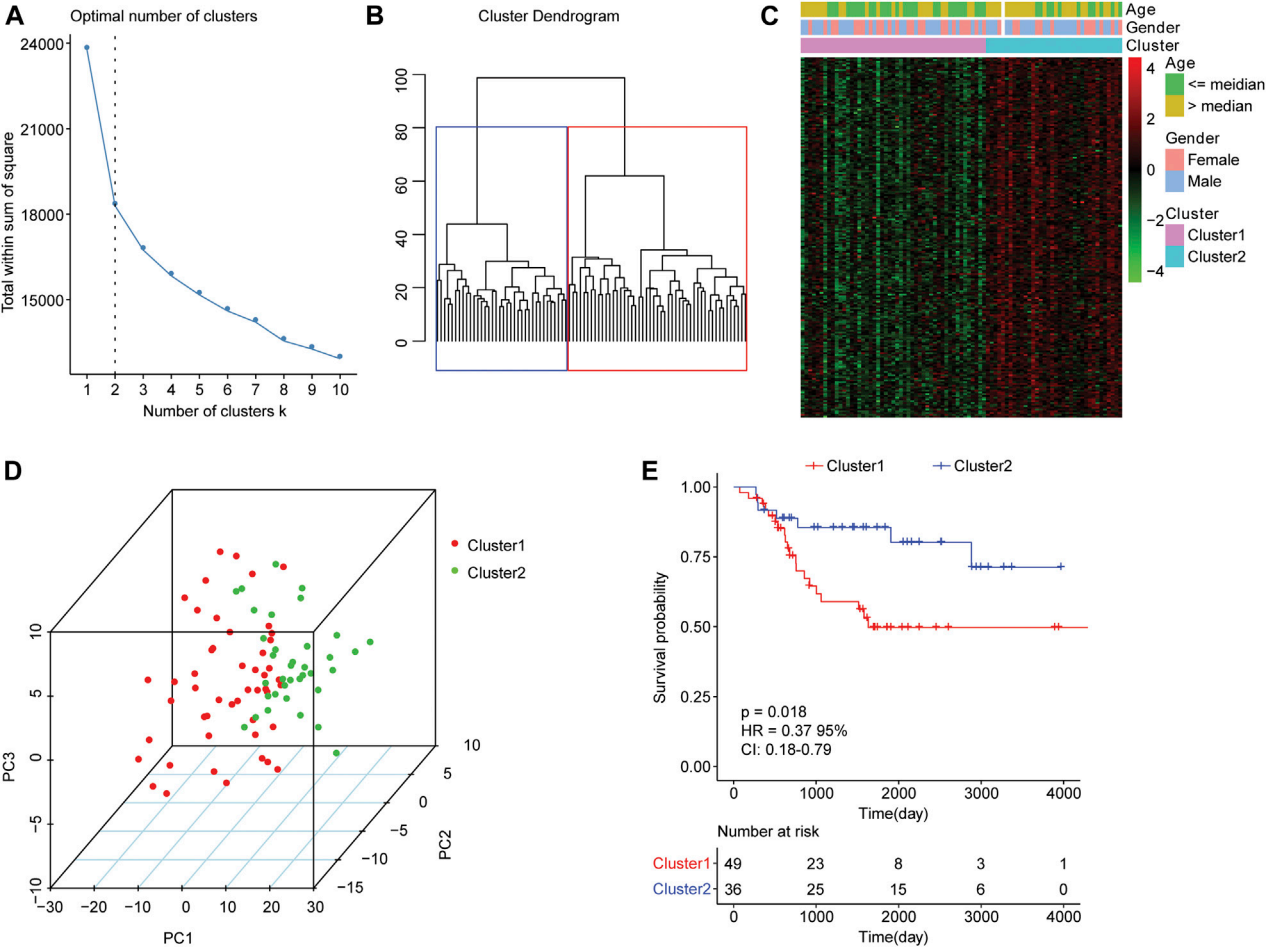
Clustering analysis of osteosarcoma samples based on mRNA levels of autophagy-related genes. **(A)** An elbow graph determined the optimal number of clusters. The horizontal axis represented the number of clusters K, and the vertical axis represented the sum of the squared errors (SSE). The point where the decline tended to be gentle was the number of the optimal cluster. **(B)** Schematic diagram of sample clustering. Different colors represented different clusters. **(C)** Heat map of the expression of autophagy-related genes in two types of samples. Behavioral genes were listed as samples. Red indicated high expression and green indicated low expression. The age and sex of the sample were marked with different colors above the heat map. **(D)** PCA analysis. The dots with different colors represented samples in different groups. The closer the dots, the more similar the expression of autophagy-related genes in the samples. **(E)** Kaplan-Meier curve. The horizontal axis represented time, the vertical axis represented survival rate, and the colors indicated different groupings. The *p* value was determined based on the log-rank test.

For the 85 osteosarcoma samples with complete survival information in the TARGET database, we used the factoextra function package in R language to perform cluster analysis based on the expression levels of 210 genes which were related to autophagy. With reference to the sum of the squared errors (SSE), we selected the number of clusters k = 2 (Figure 1A) to cluster the samples into two types. The clustering diagram (Figure 1B) and the expression calorimetry diagram (Figure 1C) showed the consistency of clustering, and these two types could be obviously distinguished. The principal component analysis (PCA) was carried out, and the result displayed that samples within cluster 1 and cluster 2 could be well distinguished (Figure 1D). Kaplan-Meier survival analysis revealed obviously worse survival outcome of osteosarcoma patients in cluster 1 than those in cluster 2 (Figure 1E). Autophagy-related genes could effectively distinguish the osteosarcoma patients with different prognosis.

### Prognostic Significance of Autophagy-Associated Genes in Osteosarcoma

**FIGURE 2:**
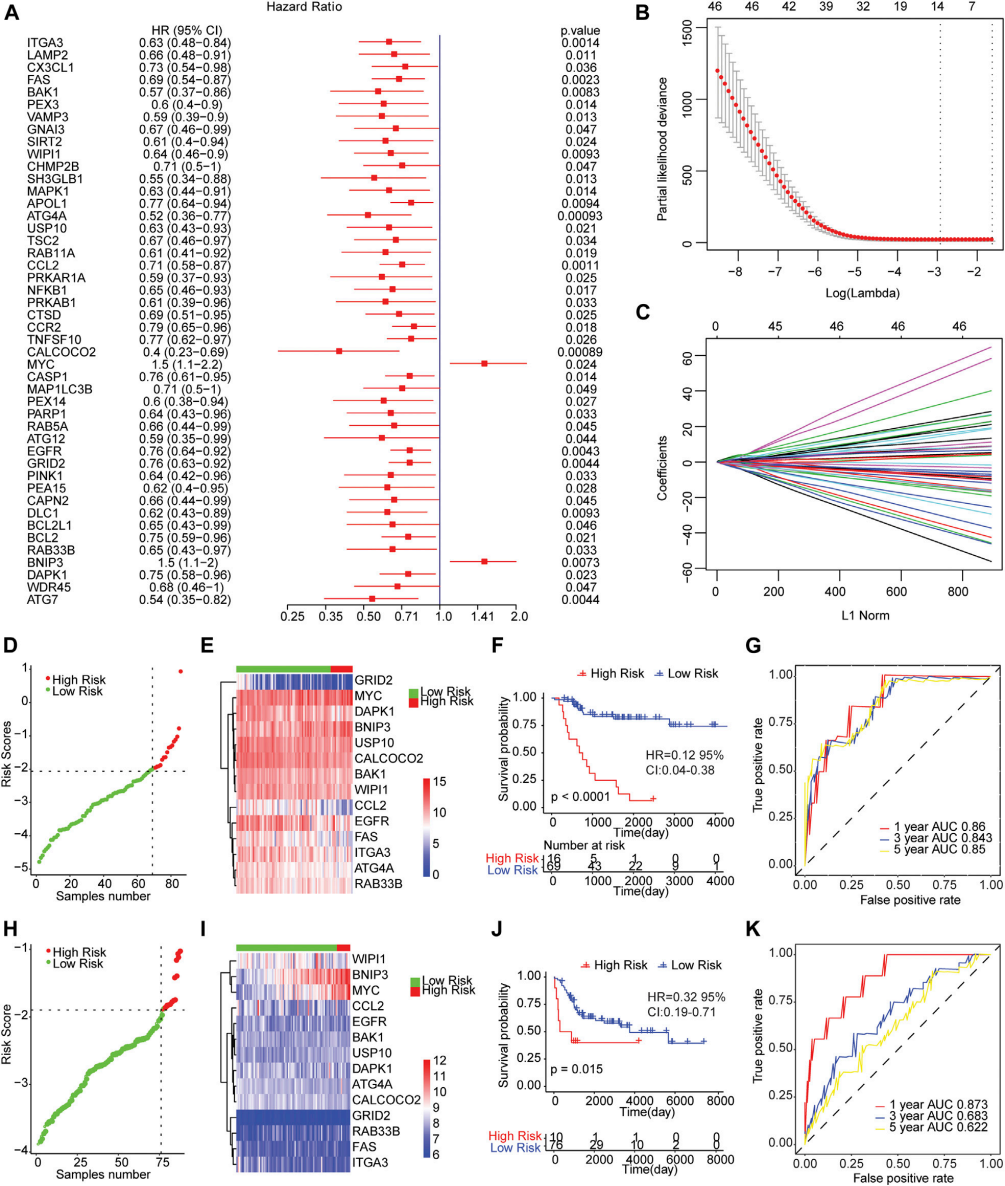
The risk score model predicted the survival of patients with osteosarcoma. **(A)** Forest map of autophagy-related genes significantly related to overall survival in univariate analysis. HR was Hazard ratio, and 95% CI was 95% confidence interval. **(B)** Diagram of the optimal number of genes in the LASSO regression model. The horizontal axis represented log (lambda), and the vertical axis represented the partial likelihood deviance. The Lambda value corresponding to the minimum value was the best. **(C)** Coefficient spectrum of LASSO Cox regression model. **(D)** The risk scores distribution of samples in the TARGET dataset. A point indicated a sample, a red point represented a sample with a higher risk score, a green point indicated a sample with a lower risk score, and the intersecting point represented the optimal risk score. **(E)** The Cluster heat map of 14 autophagy-related gene expressions in the TARGET dataset. Behavioral genes were listed as samples. Red represented high expression and blue represented low expression. Different colors indicated the sample groups above the heat map. **(F)** Kaplan-Meier survival curve of samples from TARGET dataset. The horizontal axis represented time, the vertical axis indicated survival rate, and different colors represented different groups. **(G)** The time-dependent ROC curve of samples from TARGET dataset. The horizontal axis indicated the false positive, the vertical axis represented the true positive, and the accuracy of the prediction was evaluated by AUC value (area under curve). **(H)** The distribution of risk scores of samples from integrated GEO dataset. **(I)** Cluster heat map of the expression levels of 14 autophagy-related genes from integrated GEO dataset. **(J)** Kaplan-Meier survival curve of GEO integrated dataset. **(K)** The time-dependent ROC curve of integrated GEO dataset.

We conducted the univariate cox regression analysis which has taken the expression levels of 210 genes related to autophagy as continuous variables, and computed the corresponding Hazard Ratio (HR) values. A total of 46 genes showed an obvious relationship with the OS of osteosarcoma using a *p* value of less than 0.05 as the selection criteria (Figure 2A), of which MYC (HR = 1.5, 95% CI: 1.1−2.2, *p* = 0.024) and BNIP3 (HR = 1.5, 95% CI: 1.1−2, *p* = 0.0073) were risk genes. The high expression of genes led to poor prognosis. Other genes related to prognosis were protective genes, and high expression of genes were conducive to patient prognosis.

Then, we carried out LASSO Cox regression analysis based on these 46 autophagy-related genes. The optimal number of genes was determined as 14 with reference to the minimum lambda value (Figures 2B,C). We established the risk score model after weighting the autophagy-associated genes expression and the coefficients to predict the survival outcome of osteosarcoma patients. Risk Score = 0.3316 * Expression Value of BNIP3-0.1571 * Expression Value of ATG4A-0.0114 * Expression Value of BAK1-0.192 * Expression Value of CALCOCO2-0.0457 * Expression Value of CCL2-0.0938 * Expression Value of DAPK1-0.0738 * Expression Value of EGFR-0.0734 * Expression Value of FAS-0.1609 * Expression Value of GRID2-0.0313 * Expression Value of ITGA3 + 0.2591 * Expression Value of MYC-0.0593 * Expression Value of RAB33B-0.1215 * Expression Value of USP10-0.0467 * Expression Value of WIPI1. We calculated the risk score of osteosarcoma patients from TARGET and GEO cohorts (GSE21257 and GSE16091 combined), and divided the samples into high and low risk groups with reference to respective optimal cut-off value. The distribution of risk score for the samples was shown in Figures 2D,H. Meanwhile, manifest difference in autophagy-associated gene expression between groups with distinct risk scores was observed (Figures 2E,I). Survival analysis revealed the survival outcome of osteosarcoma samples with high risk scores was worse than thoses with low risk score (Figures 2F,J). In addition, the time-dependent ROC analysis displayed that the AUC of osteosarcoma patients in TARGET dataset for 1-year, 3-years, and 5-years OS were 0.86, 0.843, and 0.85, respectively (Figure 2G), and 0.873, 0.683, and 0.622 for the GEO cohort (Figure 2K). The result indicated that the risk models in both datasets effectively predicted the prognosis of patients with osteosarcoma. Overall, those results suggested that the risk assessment models constructed based on 14 autophagy-related genes including ATG4A, BAK1, BNIP3, CALCOCO2, CCL2, DAPK1, EGFR, FAS, GRID2, ITGA3, MYC, RAB33B, USP10, and WIPI1 were able to forecast the survival outcome of osteosarcoma patients.

### Immune Infiltration Analysis

**FIGURE 3:**
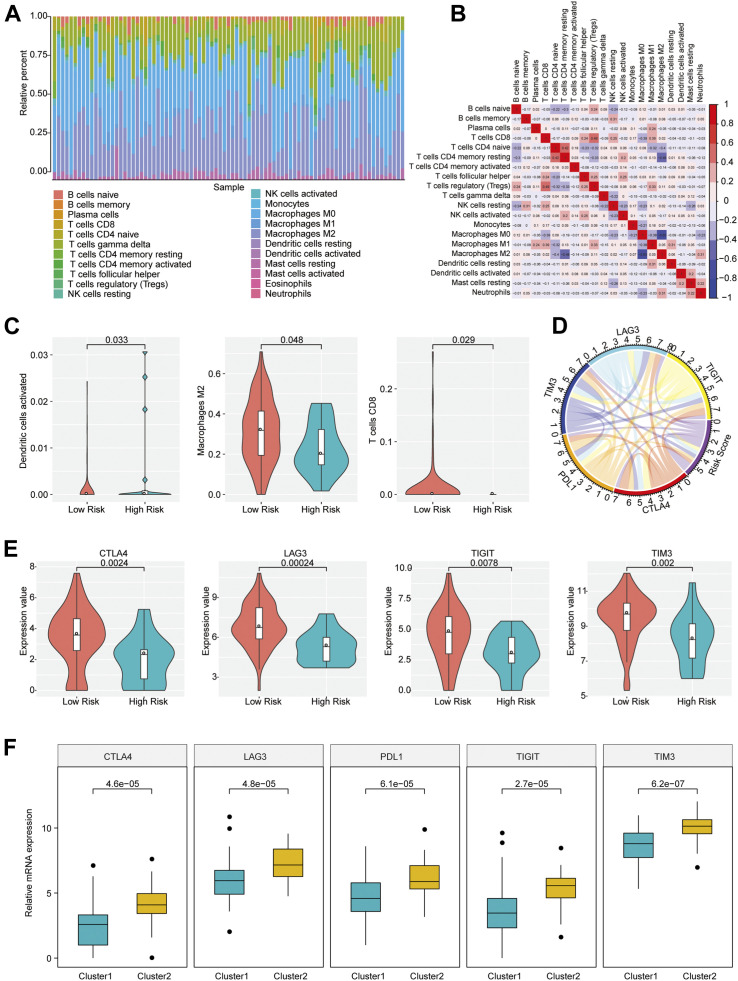
Immune infiltration of patients with osteosarcoma in high- and low-risk group. **(A)** The proportion of 22 immune infiltration cells in all patients. **(B)** Correlation matrix of the proportion of 22 immune infiltration cells. Red represented a positive correlation and blue represented a negative correlation. The darker the color, the greater the correlation. **(C)** Violin plot of immune cells with significantly different infiltration proportions in high- and low-risk groups. Different colors indicated high- and low-risk groups, and the vertical axis represented the relative infiltration proportion of different immune cells. **(D)** Circos diagram of the correlation between risk score and the expression of five key immune checkpoints. The immune checkpoints and Risk Score were represented by different colors. Cyan: LAG3; Yellow: TIGIT; Red: CTLA4; Orange: PDL1; Blue: TIM3; Purple: Risk Score. **(E)** Immune checkpoints with different expression levels in the high- and low-risk groups. **(F)** Immune checkpoints with different expression levels in different clusters.

The distinction in infiltration of the 22 immune cell types between osteosarcoma patients with high and low risk scores was analyzed by using CIBERSORT and LM22 feature matrix. The immune infiltration landscape of 85 osteosarcoma patients was shown in Figure 3A. The proportions of immune cell infiltration in different patients were different, which probably reflected the inherent features of individuals. A weak correlation of the infiltration proportion among different immune cell types was found (Figure 3B), which indicated that there was a large heterogeneity in the infiltration of different immune cells in tumor patients. Moreover, we found that activated dendritic cells, M2 macrophages and CD8 T cells had remarkable differences in the degree of infiltration between groups with different risk scores. As shown in Figure 3C, the infiltration proportions of activated dendritic cells, M2 macrophages, and CD8 T cells were significantly higher in the low risk group than those in the high risk group, which might be associated with the prognostic difference between these two groups.

Immune checkpoints have been the research hotspots in recent years, which show great clinical significance and provide promising treatment target in cancer. It was found that there was a significant relationship of the risk score with important immune checkpoints expressions (CTLA4, PDL1, TIM3, LAG3, TIGIT) in osteosarcoma patients (Figure 3D and Supplementary Table S3). In addition, five immune checkpoints expressions in high and low risk groups and different clusters of osteosarcoma patients were investigated. The result revealed that CTLA4, TIM3, LAG3, and TIGIT expression levels in the group with low risk scores were obviously elevated compared with the group with high risk scores (*p* < 0.05) (Figure 3E), and the expressions of CTLA4, PDL1, TIM3, LAG3, and TIGIT in Cluster 2 samples were strikingly increased compared with those in Cluster 1 samples (Figure 3F), indicating that the patients of the group with low risk scores and Cluster 2 were probably more sensitive to the treatment of immune checkpoint inhibitors.

### GSEA Enrichment Analysis

**FIGURE 4:**
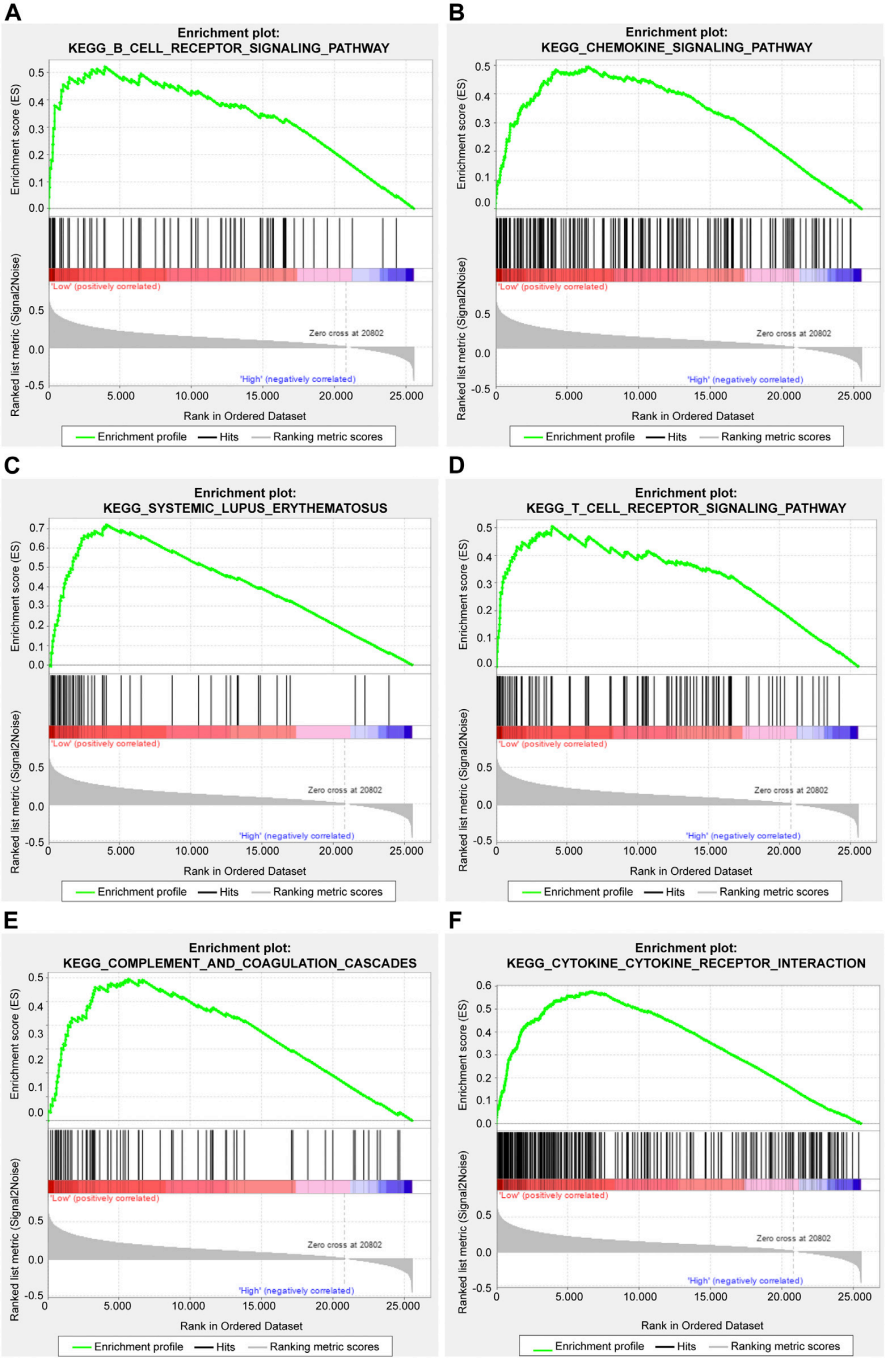
The top six significantly enriched pathways in GSEA enrichment analysis. **(A)** B cell receptor signaling pathway; **(B)** Chemokine signaling pathway; **(C)** Systemic lupus erythematosus; **(D)** T cell receptor signaling pathway; **(E)** Complement and coagulation cascades; **(F)** Cytokine cytokine receptor interaction.

The gene set enrichment analysis (GSEA) was carried out for osteosarcoma patients with different risk scores, and the significantly enriched KEGG pathway was screened with the threshold of *p* value less than 0.05. A total of 23 pathways were significantly enriched as shown in Table 2. The top six pathways were displayed in Figures 4A–F. It was found that the immune-related pathways were more likely to be enriched in osteosarcoma patients with low risk scores.

**TABLE 2:** GSEA enrichment analysis.

KEGG pathway	Normalized enrichment score	NOM p-val
KEGG_SYSTEMIC_LUPUS_ERYTHEMATOSUS	1.7960094	0.012793177
KEGG_T_CELL_RECEPTOR_SIGNALING_PATHWAY	1.7504385	0.016666668
KEGG_CYTOKINE_CYTOKINE_RECEPTOR_INTERACTION	1.7406583	0
KEGG_B_CELL_RECEPTOR_SIGNALING_PATHWAY	1.7239578	0.025806451
KEGG_COMPLEMENT_AND_COAGULATION_CASCADES	1.7081273	0
KEGG_CHEMOKINE_SIGNALING_PATHWAY	1.7065754	0.023206752
KEGG_INTESTINAL_IMMUNE_NETWORK_FOR_IGA_PRODUCTION	1.700867	0.006423983
KEGG_HEMATOPOIETIC_CELL_LINEAGE	1.6924057	0.015151516
KEGG_CELL_ADHESION_MOLECULES_CAMS	1.6835115	0.014861995
KEGG_PRIMARY_IMMUNODEFICIENCY	1.6811936	0.010845987
KEGG_ALLOGRAFT_REJECTION	1.6579281	0.031982943
KEGG_ASTHMA	1.6465044	0.02258727
KEGG_NATURAL_KILLER_CELL_MEDIATED_CYTOTOXICITY	1.6440951	0.03088803
KEGG_LEUKOCYTE_TRANSENDOTHELIAL_MIGRATION	1.6361815	0.019480519
KEGG_AUTOIMMUNE_THYROID_DISEASE	1.6160356	0.020220589
KEGG_TYPE_I_DIABETES_MELLITUS	1.6096363	0.04621849
KEGG_RENIN_ANGIOTENSIN_SYSTEM	1.5964051	0.02631579
KEGG_ENDOCYTOSIS	1.5839647	0.026373627
KEGG_GLYCOSAMINOGLYCAN_DEGRADATION	1.5492109	0.040169135
KEGG_JAK_STAT_SIGNALING_PATHWAY	1.519548	0.021400778
KEGG_FC_EPSILON_RI_SIGNALING_PATHWAY	1.4883424	0.04684318
KEGG_VASCULAR_SMOOTH_MUSCLE_CONTRACTION	1.4564767	0.046653144
KEGG_REGULATION_OF_ACTIN_CYTOSKELETON	1.4311482	0.042462844

### Risk Score Could Independently Predict the Prognosis of Osteosarcoma Patients

**FIGURE 5:**
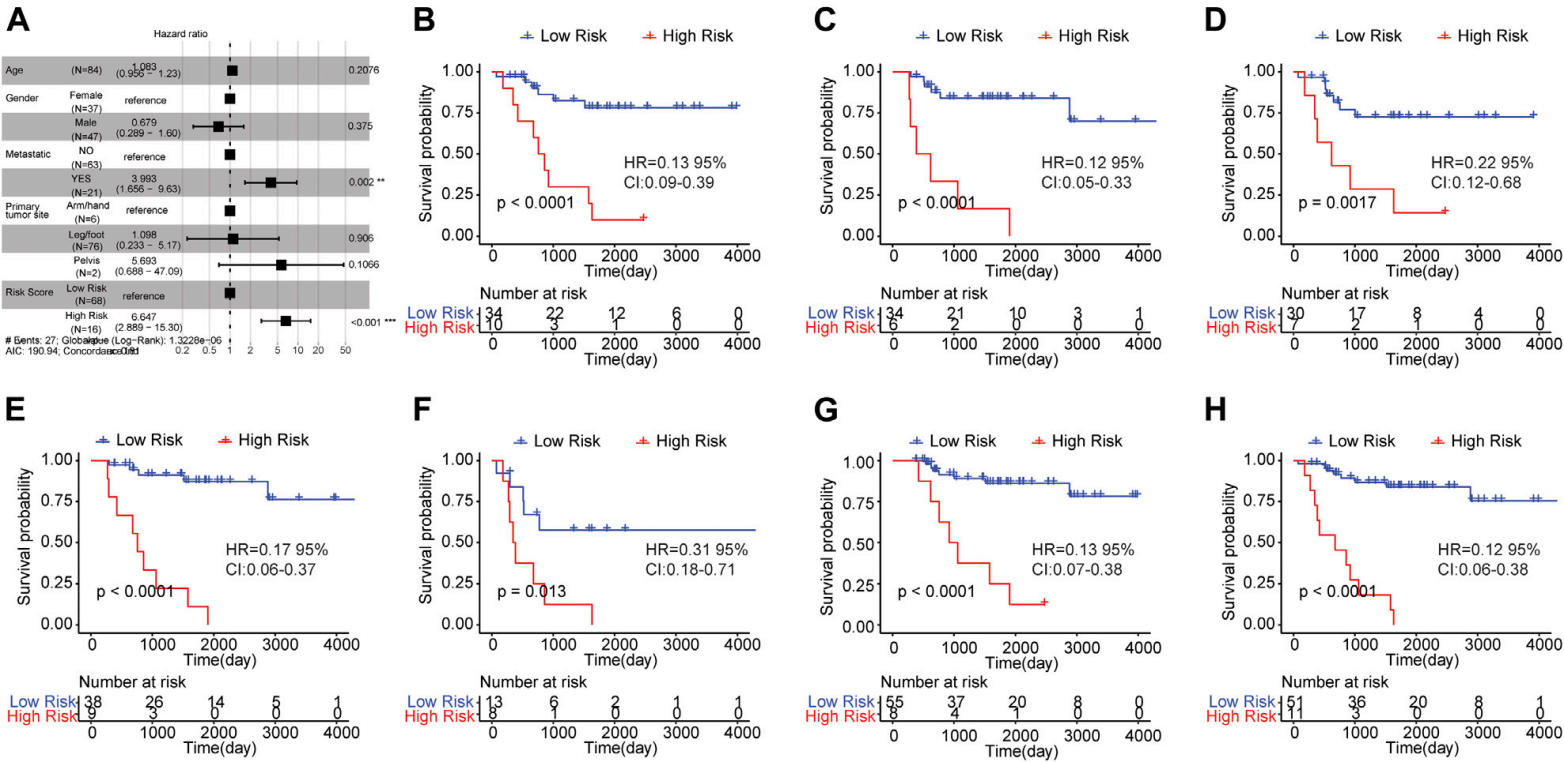
Risk score was an independent prognostic indicator for osteosarcoma. **(A)** Multi-factor Cox regression analysis of forest map. Compared with the reference sample, the sample with hazard ratio greater than one had a higher risk of death, and the sample with hazard ratio less than one had a lower risk of death. **(B**–**H)** Kaplan-Meier survival curves of osteosarcoma samples with different clinicopathological factors. The horizontal axis represented time, the vertical axis indicated survival rate, and different colors represented different groups. The *p* value was evaluated based on the log-rank test.

To verify whether risk score could independently predict the prognosis of osteosarcoma patients, we conducted the multivariate Cox regression analysis which took age, gender, metastasis, primary tumor site and risk score into account. The result was shown in Figure 5A. A significant relationship between risk score and survival outcome was still observed, and the higher the risk score, the greater the death risk, indicating that risk score was a biomarker for poor survival outcome (HR = 6.644, 95% CI: 2.883–15.31, *p* < 0.001). Besides, metastasis was also an independent prognostic factor.

Subsequently, the osteosarcoma patients were classified based on clinicopathological features (age, gender, and metastasis) and survival analysis was carried out to investigate the prognostic significance of risk score in osteosarcoma patients with distinct clinicopathological features. The samples were grouped by the median age (14) and the results showed that the OS of high risk group was worse than that of low risk group in samples with age <= 14 (Figure 5B) and age >14 (Figure 5C). Moreover, in female (Figure 5D)/male samples (Figure 5E), metastatic (Figure 5F)/non-metastatic samples (Figure 5G) and samples with leg/foot as the primary tumor location (Figure 5H), the OS of the group with high risk score was inferior in comparison to the group with low risk score. These findings revealed that risk score was a potential signature that could independently forecast the survival outcome of osteosarcoma patients.

### Nomogram Model Could Better Forecast the Survival of Osteosarcoma Patients

**FIGURE 6:**
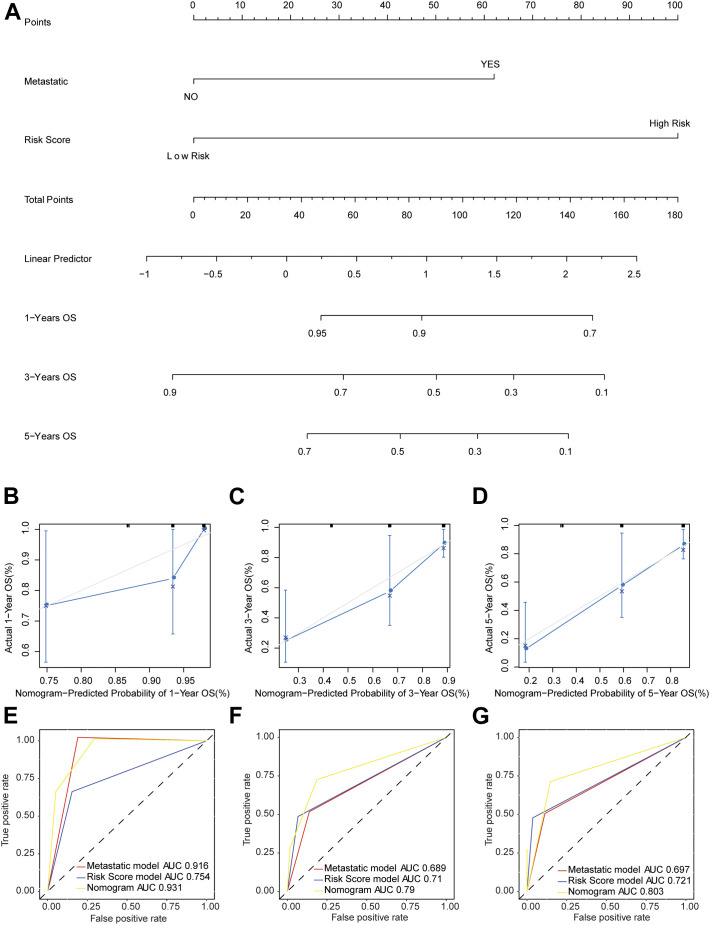
Nomogram model predicted the survival of patients with osteosarcoma. **(A)** The probability of OS in patients with osteosarcoma at 1, 3, and 5 years based on nomogram. **(B**–**D)** Calibration curve to predict the probability of OS in patients with osteosarcoma at 1, 3, and 5 years based on nomogram. The X axis represented predicted survival rate, and the Y axis represented actual survival rate. **(E**–**G)** The time-dependent ROC curve of OS in patients with osteosarcoma at 1, 3, and 5 years based on nomogram.

The nomogram model was established based on risk score and metastatic status (Figure 6A). Then, the nomogram model was verified by proportional hazards (PH) assumption, and conformed to the PH assumption test (Supplementary Figure S1). Three lines were drawn upward to measure the points of each factor in the nomogram. Subsequently, we plotted a line downward from the total points, which represented the sum of all points here, to obtain the 1-, 3-, and 5-years OS for osteosarcoma patients. The calibration curve was close to the ideal curve (gray straight line), which suggested high consistency between the predicted result and actual result (Figures 6B–D). When predicting the survival outcome of osteosarcoma patients at 1, 3, and 5 years, the AUC value of the nomogram model based on two independent factors for prognosis was higher than that based on one (Figures 6E–G), suggesting the better performance of the nomogram model in the prognostic prediction of osteosarcoma patients.

## Discussion

Osteosarcoma is a frequent malignancy in bone among children and adolescents, with an estimated incidence rate of 3 per million per year all over the world. Osteosarcoma is often accompanied with early metastasis, thus being considered as an invasive tumor [26, 27]. Although radiotherapy and neo-adjuvant chemotherapy have been developing over the past years, the OS at 5 years in metastatic cases remains only about 20%. The cure rate for osteosarcoma patients that have focal tumor increases up to 70% [28, 29]. Therefore, it is urgent to explore and identify novel prognostic biomarkers for proper clinical decision making, which may provide a searchable idea to ameliorate treatment status and survival outcome of osteosarcoma patients.

Autophagy is self-degradation targeting defective proteins and organelles, and maintains the function of mitochondria under the condition of stress. Extensive research has reveal that autophagy is associated with tumor initiation and progression. Indeed, autophagy could contribute to suppression of tumorigenesis of liver tumor through cell-intrinsic p62 accumulation [30]. Another study found that autophagy suppressed the pancreatic tumor formation by p53 loss [31]. Nevertheless, autophagy was actually required for growth, survival, and tumorigenesis of pancreatic cancers [32]. Autophagy-related genes were upregulated in breast cancer cells transformed with RAS and promoted the invasion of cancer cells [33]. Hence, autophagy provides a potential alternative for osteosarcoma treatment.

In this study, 210 autophagy-related genes were collected which could distinguish the osteosarcoma patients with significantly different prognoses. LASSO Cox regression analysis was carried out and identified 14 optimized autophagy-related genes, including ATG4A, BAK1, BNIP3, CALCOCO2, CCL2, DAPK1, EGFR, FAS, GRID2, ITGA3, MYC, RAB33B, USP10, and WIPI1, for the prognosis of osteosarcoma. ATG4A, a redox-regulated cysteine protease, is a vital autophagy regulator. ATG4A promoted the transition from epithelium to mesenchyme partly by the Notch signaling pathway in osteosarcoma cells [34] and was related to reduced risk for lung cancer [35]. Another report showed that hypomethylation of ATG4A predicted a poor prognosis for ovarian cancer patients [36]. BAK1, a member of the B cell lymphoma family containing BH3 domain, could induce the mitochondria-mediated apoptosis by interacting with other proteins. Studies demonstrated that BAK1 played a role in drug resistance and tumor proliferation in many cancers including breast, lung and cervical cancers [37–39]. An established prognostic signature based on seven genes including BAK1 was able to predict the survival outcome of head and neck squamous cell carcinoma patients [40]. BNIP3, also a member of B cell lymphoma family, could regulate the cell survival, autophagy, and cytoprotection. Moreover, research suggested that the elevated BNIP3 levels were correlated with progression to metastasis and poor prognosis in multiple cancers, such as breast and lung cancers, and uveal melanoma [41–43]. BNIP3 overexpression could induce the apoptosis of osteosarcoma cells, and BNIP3 inhibition plays a suppressive role in osteosarcoma cells apoptosis [44, 45]. CALCOCO2, also known as nuclear domain 10 protein 52, is implicated in autophagy factors recruitment and TANK-binding kinase 1 (TBK1) activation [46]. The model based on 16 autophagy related genes including CALCOCO2 could discriminate the multiple myeloma patients with distinct clinical outcomes, presenting potential prognostic value in multiple myeloma research [47]. CCL2 belongs to the CC chemokine family and is secreted by various cells including endothelial cells, fibroblasts, monocytes and tumor cells [48]. A previous study indicated that CCL2 could promote the invasion of pancreatic ductal adenocarcinoma [49] and the metastasis in cervical cancer [50]. In addition, high levels of CCL2 were related to the inferior survival outcome in gastric cancer [51]. Compared with the low-grade osteosarcoma, CCL2 expression was elevated in the osteosarcoma with high grade, which enhanced the proliferative and invasive abilities of osteosarcoma cells [52]. DAPK1 belongs to the Ser/Thr kinase family and is considered as a key regulator of autophagy and apoptosis [53]. It was found that DAPK1 expression could significantly inhibit the tumor growth and metastasis [54]. Down-regulation of DAPK1 expression may be a prognostic factor in many tumors, such as diffuse large B-cell lymphoma [55] and liver cancer [56]. EGFR is a receptor tyrosine kinase (RTK) for ErbB family, and exhibits over-expression in various tumor cells [57]. Overexpression of EGFR is related to survival, invasion, metastasis, drug resistance, and poor prognosis of tumor [58], for example, EGFR is considered as an indicator of inferior prognosis in node-negative breast cancer [59]. EGFR was reported to be abnormally expressed in osteosarcoma, and the expression as well as amplification of EGFR were observed in the osteosarcoma with high grade (PMID: [60, 61]). FAS is an essential enzyme in the process of lipogenesis, and could effectively maintain the energy homeostasis. FAS expression level is up-regulated in several cancers and exhibits a strong effect on tumor cell proliferation and apoptosis. It was found that inhibition of FAS could obviously inhibited the capacity of growth and migration of bladder cancer cells [62], and Fas was proved to be a significant marker for the prognosis of breast cancer [63]. GRID2, belongs to the ionic glutamate receptor family, and regulates excitatory synaptic transmission [64]. Previous study has confirmed that GRID2 was related to the inferior survival outcome of prostate and gastric cancers [65, 66]. ITGA3 belongs to the integrin family of cell surface receptors and is involved in the survival, proliferation, and migration of cells. In gastric carcinomas, ITGA3 expression could facilitate the invasion [67] and was considered as a key signature for colon cancer [68]. Interestingly, ITGA3 was also a component of the prognostic signature for head and neck squamous cell carcinoma, with a similar role like BAK1, as described above [40]. ITGA3 polymorphisms might influence the osteosarcoma in terms of the incidence rate, metastatic status and prognosis, which was considered as a potential signature for osteosarcoma [69]. MYC, an oncogenic transcription factor, regulates cell proliferation, apoptosis, and carcinogenesis [70]. Research revealed that mutations of c-MYC could result in tumorigenesis [71]. The amplification of c-MYC was observed during the development of hepatocellular carcinoma, which was associated with impaired survival [72]. Moreover, increased expression of c-MYC was proved to enhance the invasive ability of osteosarcoma cells by targeting MEK-ERK pathway [73]. RAB33B belongs to the Rab family of small GTP binding proteins, and regulates the fusion of autophagosomes and membrane trafficking [74]. In addition, RAB33B was identified as a biomarker for lung cancer diagnosis [75]. However, its prognostic value or relationship with the survival outcome of cancer is rarely reported. USP10 belongs to the ubiquitin-specific protease family, modulates DNA damage response and autophagy [76, 77]. Moreover, USP10 inhibited cell growth and invasion in lung cancer [78], and was an independent factor for the prognosis of gastric carcinoma [79]. WIPI1, a member of WD-repeat protein which interacts with phosphoinositides (WIPI) family, participates in the formation of autophagosome [80]. Furthermore, WIPI1 was a relevant novel melanoma marker [81], and the increased expression of WIPI1 indicated poor clinical outcome in uveal melanoma [82] In osteosarcoma, WIPI1 expression was obviously elevated, which promoted the proliferation of osteosarcoma cells through regulating CDKN1A expression [83]. This research further confirmed the potential prognostic value of the identified genes in osteosarcoma.

Several prognostic models of osteosarcoma have been established in previous research. For example, Qu et al. constructed a 5-gene-signature for the prognosis prediction of osteosarcoma based on the super-enhancer-associated genes [84] The model basing on the biomarkers including RBC, PNI, CRE, Ca^2^⁺and LSR in blood presented good performance in predicting the overall survival of osteosarcoma patients [85]. Lin et al. established a predictive model with five differentially expressed genes related to metastasis between the metastatic and non-metastatic samples for the prognosis of osteosarcoma patients [86]. To our knowledge, we are the first to construct a prognostic model of osteosarcoma with autophagy related genes *via* integrated methods of bioinformatics and machine learning.

Except for risk score, metastasis was also an independent factor for osteosarcoma prognosis. We applied risk score and metastasis as independent prognostic factors to construct a nomogram model for OS prediction in osteosarcoma patients. The result indicated that the nomogram model with two independent factors showed better performance in OS prediction than that with one factor. Furthermore, three immune cell types presented obvious distinction in infiltration proportion between samples with high and low risk scores. The infiltration proportions of activated dendritic cells, M2 macrophages and CD8 T cells were significantly higher in the low risk group than those in the high risk group. Zhang et al. indicated that the osteosarcoma patients with superior survival outcome had higher levels of M2 macrophages, compared with those with inferior survival outcome [87], which was consistent with our finding that the low-risk osteosarcoma patients with improved prognosis had higher proportion of M2 macrophage. Gomez-Brouchet et al. found that CD8 T cells were related to the non-metastatic osteosarcoma [88], and higher infiltration rate of CD8 T cells indicated improved survival outcome [89], showing consistency with our result that the low-risk osteosarcoma patients with superior prognosis had higher infiltration proportion of CD8 T cells. In terms of activated dendritic cells, Wang et al. demonstrated that activated dendritic cell was an independent predictor of osteosarcoma and the model basing on several immune cell types including activated dendritic cell could reliably predict the prognosis of osteosarcoma patients [90], suggesting the potentially important role of activated dendritic cells in osteosarcoma. However, the specific functions of these significantly distinct infiltrating immune cells between the osteosarcoma patients with high and low risk scores and the underlying mechanism still need further research. In addition, analysis of five immune checkpoints in osteosarcoma patients showed that the expressions of CTLA4, TIM3, LAG3, and TIGIT in the samples with low risk scores were markedly elevated compared with those with high risk scores, suggesting that patients with low risk scores might be sensitive to the treatment targeting immune checkpoints.

However, there are some limitations in our study. First, it lacks experimental work, which would be helpful for further validation of the results. Second, the sample size is relatively small. Further research with more samples is needed to better evaluate the performance of the model and elucidate the underlying mechanism in the future.

## Conclusion

In brief, we identified a 14-autophagy-gene-based prognostic signature in osteosarcoma. Based on these 14 genes associated with autophagy, a model was established and risk score was able to predict the prognosis of osteosarcoma patients independently. Importantly, a nomogram model based on risk score and metastasis was established and exhibited better performance to predict the OS at 1, 3, and 5 years for osteosarcoma patients.

## Data Availability

The original contributions presented in the study are included in the article/Supplementary Material, further inquiries can be directed to the corresponding author.

## Supplementary Material

Supplementary Figure 1 Schoenfeld residual plot of proportional hazards (PH) assumption. The abscissa represented the time, and the ordinate represented the Beta value of each variable. The solid line was the fitted smooth curve, and the dotted line was the standard deviation of 2 units above and below the fitted curve. If the curve deviated by 2 units of standard deviation, then the proportional risk assumption was not satisfied.
