# Supplementary figures and images for: Prognostic Signature of Osteosarcoma Based on 14 Autophagy-Related Genes
Source: Pathol Oncol Res. 2021 Jul 16;27:1609782. doi: 10.3389/pore.2021.1609782 (PMC8322075; doi:10.3389/pore.2021.1609782)

Schoenfeld Individual Test p: 0.0597

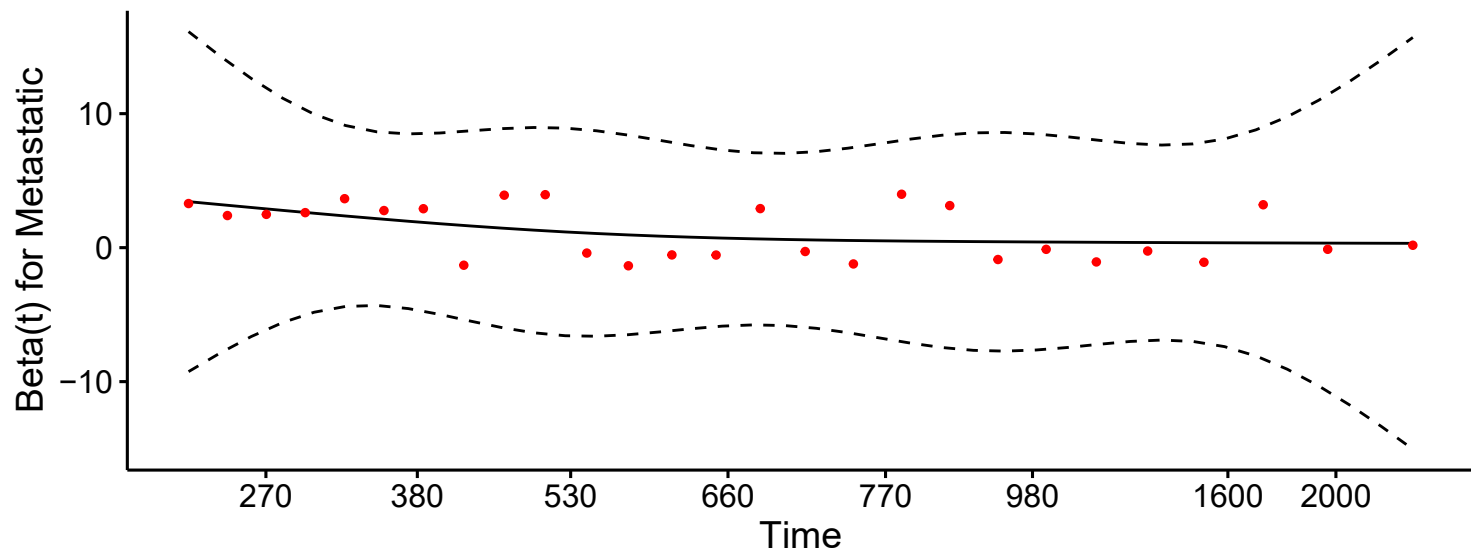

Schoenfeld Individual Test p: 0.1794

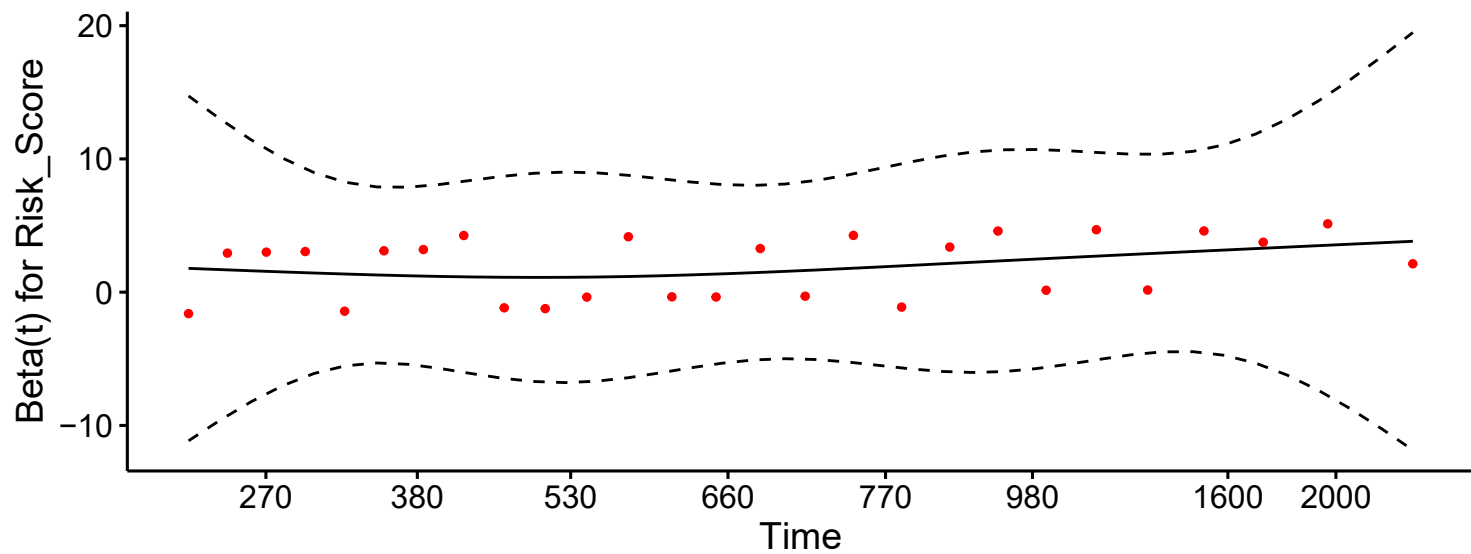

Supplement: Supplementary file 4 [file Image1.pdf]
